# Line-Dependent Differences in Volatile Flavor Profiles of Zao-Po Vinegar Wenchang Chicken: An Integrated E-Nose, HS-SPME-GC–MS, and HS-GC–IMS Analysis

**DOI:** 10.3390/foods15193464

**Published:** 2026-09-28

**Authors:** Haokai Fan, Jun Wang, Zhe Liu, Tieshan Xu, Qicheng Jiang, Meiling Chen, Xinli Zheng, Zhemin Lin, Lihong Gu, Zhengguang Wang

**Affiliations:** 1 Hainan Institute, Zhejiang University, Sanya 572000, China; 2 College of Animal Sciences, Zhejiang University, Hangzhou 310058, China; 3 Institute of Animal Science and Veterinary Medicine, Hainan Academy of Agricultural Sciences, Haikou 571199, China; 4 Tropical Crops Genetic Resources Institute, Chinese Academy of Tropical Agricultural Sciences, Haikou 571101, China; 5 College of Animal Science and Technology, China Agricultural University, Beijing 100193, China

**Keywords:** volatile organic compounds (VOCs), flavor profile, correlation analysis, biochemical parameters, antioxidant parameters

## Abstract

Wenchang chicken is a famous indigenous breed from Hainan Province, China, and its characteristic flavor is a key determinant of product quality and consumer acceptance. However, differences in volatile flavor profiles among Wenchang chicken lines under standardized rearing and processing conditions remain insufficiently characterized. In this study, an electronic nose (E-nose), headspace solid-phase microextraction coupled with gas chromatography–mass spectrometry (HS-SPME-GC–MS), headspace gas chromatography–ion mobility spectrometry (HS-GC–IMS), biochemical and antioxidant analyses, and Pearson correlation analysis were employed to comprehensively evaluate Zao-Po vinegar Wenchang chicken prepared from twelve Wenchang chicken lines, with three biological replicates per line (n = 3 per line; 36 birds in total). E-nose analysis showed clear separation among different lines, with the first two principal components explaining 97.36% of the total variance. HS-SPME-GC–MS identified 85 volatile organic compounds belonging to 11 chemical classes, of which 14 showed significant line-associated differences after Benjamini–Hochberg false discovery rate (FDR) correction (FDR-adjusted *p* < 0.05), with decanal, octanal, nonanal, and pentanal among the representative discriminatory VOCs. Pearson correlation analysis, with Benjamini–Hochberg FDR correction for multiple testing, further revealed significant associations between selected biochemical and antioxidant parameters and individual VOCs. These findings suggest potential associations between line-level physiological characteristics and variation in volatile profiles. These results provide a basis for further investigation of the biological mechanisms underlying flavor variation and for the evaluation and utilization of Wenchang chicken germplasm.

## 1. Introduction

Chicken meat is one of the most widely consumed sources of animal protein worldwide and is highly valued by consumers for its nutritional value and desirable eating quality [1]. Wenchang chicken is a well-known indigenous chicken breed originating from Hainan Province, China. It is highly reputed nationwide for its tender meat, rich flavor, and fatty but non-greasy skin. Owing to its exceptional eating quality, it ranks first among Hainan’s “Four Famous Dishes” [2].

Zao-Po vinegar is a fermented food highly favored by consumers. It contains abundant amino acids, phenolic compounds, organic acids, and beneficial microorganisms [3]. During the preparation of Zao-Po vinegar Wenchang chicken, organic acids, amino acids, and other flavor-active compounds from Zao-Po vinegar interact with the abundant lipids and endogenous flavor precursors present in Wenchang chicken. These components undergo a series of complex reactions, including lipid oxidation, Maillard reactions, degradation of sulfur-containing precursors, and fermentation-associated metabolism, resulting in the formation of a characteristic aroma profile combining sour, meaty, and fermented notes. Moreover, Maillard reaction intermediates can further react with lipid oxidation products, particularly aldehydes and other carbonyl compounds, generating additional aroma compounds and further increasing the complexity of meat flavor [4].

Although the processing conditions play an important role in the generation of volatile compounds, line-associated variation in muscle composition and metabolic characteristics may contribute to differences in meat flavor profiles. Previous studies have demonstrated that different chicken lines exhibit considerable variation in intramuscular lipid content, fatty acid composition, amino acid profiles, and antioxidant capacity, all of which may influence the availability of flavor precursors and the formation of VOCs. Therefore, even under standardized processing conditions, different Wenchang chicken lines may produce distinct volatile flavor profiles associated with differences in precursor metabolism and subsequent aroma formation [1,4].

Previous studies have demonstrated that chickens with different selection histories and production objectives exhibit substantial differences in growth performance, carcass composition, muscle characteristics, and meat quality. Mueller et al. [5] directly compared broiler-, layer-, and dual-purpose chicken types and reported marked differences in growth rate, carcass characteristics, breast meat proportion, and several meat quality traits. Similarly, Sandercock et al. [6] demonstrated substantial variation in body size, carcass and muscle yields, abdominal fat deposition, muscle quality, and sensory characteristics among commercial broiler, layer, and traditional chicken lines. Differences in breast yield, muscle pH and color, muscle structure, and lipid oxidation have also been reported between dual-purpose and commercial broiler chickens [7]. Importantly, such variation extends to flavor-related precursors and metabolites. Shin et al. [8] reported significant breed-related differences in IMP and AMP contents, free amino acid profiles, fatty acid composition, and VOC profiles among three Korean native chicken breeds. Similarly, differences in amino acid composition and volatile compounds have been observed between indigenous and commercial broiler chickens [9], while distinct volatile flavor profiles have also been reported among Chinese indigenous chicken breeds [10]. Collectively, these findings indicate that differences in selection history and production objectives may be accompanied by variation in muscle composition and flavor precursor profiles, providing a biological basis for line-associated differences in volatile flavor profiles among Wenchang chickens.

Although electronic noses (E-noses), GC–MS, and GC–IMS have been widely applied in meat flavor research, the volatile flavor characteristics of Zao-Po vinegar Wenchang chicken prepared from different Wenchang chicken lines have not been systematically investigated. We hypothesized that, under standardized rearing and processing conditions, Wenchang chicken lines would exhibit distinct volatile flavor profiles associated with line-related differences in flavor precursor composition and metabolism. Therefore, the present study systematically investigated the volatile flavor characteristics of Zao-Po vinegar Wenchang chicken prepared from 12 Wenchang chicken lines under standardized processing conditions. The overall aroma characteristics of different chicken lines were first compared using an E-nose, followed by HS-SPME-GC–MS for comprehensive characterization of volatile compounds and HS-GC–IMS for volatile fingerprint profiling. In addition, biochemical and antioxidant-related parameters were determined, and correlation analysis was performed to explore their associations with individual volatile compounds and to identify potential physiological factors associated with line-dependent variation in volatile flavor profiles. The findings of this study are expected to provide a theoretical basis for Wenchang chicken germplasm evaluation and utilization, flavor quality improvement, and the standardized production of Zao-Po vinegar Wenchang chicken.

## 2. Materials and Methods

### 2.1. Sample Collection

Fresh Wenchang chickens representing 12 breeding lines were used in this study. The birds were obtained from Hainan Chuanwei Wenchang Chicken Industry Co., Ltd., Wenchang Longquan Wenchang Chicken Industrial Co., Ltd., Hainan (Tanniu) Wenchang Chicken Co., Ltd., and Wenchang Fu Qirui Breeding Farm, all located in Wenchang, China. All lines belonged to the Wenchang chicken breed but originated from breeding populations maintained by different breeding enterprises in Hainan Province. The lines had undergone long-term artificial selection under different breeding programs and were classified according to their primary selection objectives as egg-production lines (M, J, W, Z1, and Z2), meat-production lines (T, U3, U4, and Y), and dual-purpose lines (U1, U2, and B). Despite their common breed origin, differences in selection history and breeding objectives may have contributed to differentiation among these lines.

To minimize environmental effects, chickens from all lines were reared at Haikou Longquan Wenchang Chicken Industrial Co., Ltd. (Haikou, China), under standardized management conditions in accordance with the geographical indication product standard for Wenchang chicken (DB 46/T 545-2021) [11]. All birds used in this study were male and were reared to 135 days of age. At the end of the rearing period, three healthy birds with uniform body weight were randomly selected from each line as biological replicates (n = 3 per line; 36 birds in total), with live body weights ranging from 1.8 to 2.0 kg. After a 12-h overnight fast, the birds were electrically stunned and slaughtered by exsanguination. Following slaughter, the pectoralis major muscles were collected and immediately used for the preparation of Zao-Po vinegar Wenchang chicken as described in Section 2.2. Detailed information on the chicken lines, selection objectives, and sources is provided in Table S3. Blood samples were collected during exsanguination.

### 2.2. Preparation of Zao-Po Vinegar Wenchang Chicken Samples

Fresh Wenchang chickens were slaughtered, and the pectoralis major muscles were collected as experimental materials. Three birds from each Wenchang chicken line were selected as biological replicates.

Zao-Po vinegar was purchased from Hainan Puqian Zao-Po Vinegar Co., Ltd. (Haikou, China), and complied with the Hainan Provincial Food Safety Standard (DBS46/005-2025). The pectoralis major muscles were cut into approximately 3 × 3 × 3 cm portions before cooking. The cooking broth was prepared by mixing Zao-Po vinegar with distilled water at a volume ratio of 1:2 (*v*/*v*). After the broth reached a boil, chicken breast samples were added at a meat-to-broth ratio of 1:5 (*w*/*v*) and cooked under gentle boiling conditions for 15 min. During cooking, the internal temperature of the chicken meat was monitored using a digital probe thermometer and confirmed to exceed 75 °C.

After cooking, the samples were immediately removed from the broth and allowed to cool naturally to room temperature. The cooked breast meat was minced, thoroughly homogenized, and immediately subjected to electronic nose (E-nose), headspace solid-phase microextraction gas chromatography–mass spectrometry (HS-SPME-GC–MS), and headspace gas chromatography–ion mobility spectrometry (HS-GC–IMS) analyses. All samples were prepared using standardized Zao-Po vinegar formulations, solid-to-liquid ratios, and cooking conditions to ensure experimental consistency.

### 2.3. Determination of Biochemical and Antioxidant Parameters

For each Wenchang chicken line, the same three individuals used for volatile flavor analysis were selected as biological replicates. Blood samples were collected, and serum was separated by centrifugation at 3000× *g* for 10 min at 4 °C, then stored at −80 °C until analysis.

Serum biochemical and antioxidant parameters, including albumin (ALB), aspartate aminotransferase (AST), total cholesterol (CHO), glucose (GLU), high-density lipoprotein cholesterol (HDL), total protein (TP), malondialdehyde (MDA), and total antioxidant capacity (T-AOC), were determined using commercial assay kits (Wuhan Servicebio Biotechnology Co., Ltd., Wuhan, China) according to the manufacturer’s instructions. The absorbances for antioxidant parameters were recorded using a microplate reader at specific wavelengths.

### 2.4. Electronic Nose (E-Nose) Analysis

Approximately 9.00 ± 0.01 g of each Zao-Po vinegar Wenchang chicken breast sample was transferred into a 40 mL headspace vial and allowed to equilibrate at room temperature for approximately 1 h before E-nose analysis. Clean air was used as the carrier gas. The sampling time was 60 s at a flow rate of 1 L min^−1^, followed by sensor cleaning for 10 s at 3 L min^−1^. Each biological sample was measured twice as technical replicates. Volatile profiles were analyzed using an ISENSO SuperNose electronic nose system (ISENSO Technologies, Shanghai, China). The response characteristics of the E-nose sensors are provided in Table S2. The maximum response value of each sensor was selected as the characteristic signal to construct the original data matrix, which was subsequently subjected to principal component analysis (PCA).

### 2.5. Headspace Solid-Phase Microextraction Gas Chromatography–Mass Spectrometry (HS-SPME-GC–MS)

Three grams of each sample were transferred into a 20 mL headspace vial. Subsequently, 1 μL of 2-methyl-3-heptanone (0.812 μg mL^−1^) was added as the internal standard. Samples were equilibrated at 60 °C for 10 min, followed by extraction using a 75 μm CAR/PDMS fiber (Supelco, Bellefonte, PA, USA) at the same temperature for 30 min. Prior to extraction, the fiber was conditioned at 250 °C for 1 h according to the manufacturer’s instructions.

Following extraction, the fiber was immediately inserted into the GC injection port and thermally desorbed for 5 min in splitless mode at an injector temperature of 250 °C. Volatile compounds were analyzed using a TSQ 9000 triple quadrupole GC–MS system (Thermo Fisher Scientific, Waltham, MA, USA). Chromatographic separation was performed on an HP-5MS capillary column (30 m × 0.25 mm × 0.25 μm). The oven temperature program was as follows: 40 °C for 4 min, which was increased to 100 °C at 4 °C min^−1^ and held for 5 min, followed by heating to 200 °C at 4 °C min^−1^ and holding for 2 min. Helium was used as the carrier gas at a constant flow rate of 1.0 mL min^−1^.

Mass spectrometric detection was performed in electron ionization (EI) mode at 70 eV, with the ion source maintained at 230 °C. Mass spectra were acquired in full-scan mode over the range of *m*/*z* 30–500. Volatile compounds were tentatively identified by comparing their mass spectra with the NIST mass spectral library using NIST MS Search Version 2.4 (National Institute of Standards and Technology, Gaithersburg, MD, USA). Only compounds with a spectral similarity index (SI) ≥ 800 were retained for subsequent analysis. Retention indices were not calculated because an n-alkane series was not analyzed under the same chromatographic conditions. Accordingly, the reported compound assignments were regarded as tentative identifications based primarily on mass-spectral library matching.

Semi-quantitative analysis was performed using 2-methyl-3-heptanone as the internal standard. The estimated concentration of each VOC was calculated according to the following equation:

Estimated concentration (μg/kg) = (A_VOC/A_IS) × (m_IS/m_sample) × 1000

where A_VOC is the peak area of the target volatile compound, A_IS is the peak area of the internal standard (2-methyl-3-heptanone), m_IS is the amount of internal standard added to the sample (μg), and m_sample is the sample mass (g).

No dedicated blank or pooled quality-control (QC) samples were included in the analytical sequence.

### 2.6. Headspace Gas Chromatography–Ion Mobility Spectrometry (HS-GC–IMS)

GC-IMS analysis was conducted using an HP-5 non-polar column (30 m × 0.32 mm, 0.25 μm), with an inlet temperature of 200 °C. The temperature program started at 45 °C, which was held for 12 min, and then increased to 130 °C at 3 °C/min, followed by an increase to 180 °C at 8 °C/min (held for 2 min). The column flow rate was 1 mL/min, with a total cycle time of 60 min. IMS conditions were set with a 5.3 cm drift tube at 45 °C, using high-purity nitrogen (≥99.999%) as the drift gas at a flow rate of 150 mL/min. For headspace sampling, 3 g of each chicken sample was placed in a 20 mL headspace vial and incubated at 60 °C for 15 min. Subsequently, 300 μL of the headspace gas was withdrawn using a heated syringe maintained at 85 °C and directly injected into the GC–IMS system for analysis.

Volatile compounds detected by HS-GC–IMS were identified using VOCal software (Version 0.4.03; G.A.S., Dortmund, Germany) based on both the retention index (RI) and ion mobility drift time, in combination with the built-in NIST 2020 GC retention index database and IMS drift time database. Three-dimensional spectra, two-dimensional topographic plots, and Gallery Plot fingerprint maps were generated using VOCal software to compare the volatile flavor profiles of Zao-Po vinegar Wenchang chicken prepared from different Wenchang chicken lines.

### 2.7. Statistical Analysis

All statistical analyses were performed using R software (Version 4.4.1; R Foundation for Statistical Computing, Vienna, Austria). Each individual bird was considered an independent experimental unit, with three biological replicates per Wenchang chicken line (n = 3 per line; 36 birds in total).

Principal component analysis (PCA) of the E-nose data was performed using the maximum response values of the sensors. Hierarchical clustering analysis (HCA) was performed on column-wise Z-score-normalized sensor responses using Euclidean distance and complete linkage.

Differences in biochemical and antioxidant parameters among Wenchang chicken lines were evaluated using one-way analysis of variance (ANOVA), followed by Duncan’s multiple range test. Pearson correlation analysis was performed between biochemical and antioxidant parameters and VOCs using line-level mean values (n = 12 lines). To account for multiple comparisons, *p* values from all parameter–VOC correlations were adjusted using the Benjamini–Hochberg FDR procedure, and correlations with FDR-adjusted *p* < 0.05 were considered statistically significant.

VOC abundance data obtained by HS-SPME-GC–MS were log10-transformed before statistical analysis. Overall differences in individual VOCs among Wenchang chicken lines were assessed using Welch’s one-way ANOVA. To account for multiple testing across the 85 identified VOCs, *p* values were adjusted using the Benjamini–Hochberg false discovery rate (BH-FDR) procedure. VOCs with FDR-adjusted *p* < 0.05 were considered statistically significant. For VOCs showing significant overall differences, pairwise comparisons among chicken lines were subsequently performed using the Games–Howell post-hoc test. All statistical analyses were performed using.

## 3. Results

### 3.1. Electronic Nose Analysis of Zao-Po Vinegar Wenchang Chicken Prepared from Different Wenchang Chicken Lines

To evaluate differences in the overall odor characteristics of Zao-Po vinegar Wenchang chicken prepared from different Wenchang chicken lines, an electronic nose combined with principal component analysis (PCA) and hierarchical cluster analysis (HCA) was employed. As shown in Figure 1, PC1 and PC2 explained 94.73% and 2.63% of the total variance, respectively, accounting for a cumulative variance of 97.36%, indicating that the electronic nose sensor array effectively captured the overall odor information of the samples.

The PCA score plot showed that the samples were distributed primarily along the PC1 axis. Specifically, the M, J, W, Z1, and Z2 lines were mainly located on the positive side of PC1, whereas the T, U1, U2, B, U3, U4, and Y lines were mainly located on the negative side. The M and T lines were positioned toward opposite ends of the PC1 axis, indicating contrasting E-nose response patterns between these two lines. Biological replicates from the same line generally showed similar distributions in the PCA score plot.

The HCA heatmap further illustrated the differences in electronic nose response patterns among the Wenchang chicken lines (Figure 2). The heatmap was generated using column-wise Z-score normalization of the sensor responses, in which red indicates a sensor response higher than the average across all chicken lines, white indicates a response close to the average, and blue indicates a response lower than the average. Therefore, the color scale reflects the relative response intensity of each sensor rather than its absolute response value.

Overall, the fourteen electronic nose sensors exhibited varying response patterns among the different chicken lines, suggesting line-associated variation in overall E-nose response patterns among the Wenchang chicken lines. Among the tested lines, M, J, Z1, W, and Z2 generally exhibited higher standardized responses across most sensors, whereas T, U4, U2, Y, U1, U3, and B showed relatively lower responses, indicating noticeable variation in the overall volatile aroma profiles among the different Wenchang chicken lines.

With respect to individual sensors, sensor_11, which is highly sensitive to volatile organic compounds (VOCs), together with sensor_12, which is mainly responsive to alkanes and certain esters, and sensor_13, which is primarily sensitive to long-chain alkanes and methyl-containing volatile compounds, exhibited relatively stronger responses in the M, J, Z1, W, and Z2 lines. These observations suggest relatively stronger sensor responses associated with VOCs, alkanes, and ester-related volatile compounds in these chicken lines. In addition, sensor_6, which is sensitive to alcohols, aldehydes, ketones, and ethers, displayed pronounced variation among chicken lines, indicating differences in sensor responses associated with these classes of volatile compounds. Likewise, sensor_4, which is sensitive to sulfur-containing compounds, and sensor_5, which is sensitive to nitrogen-containing compounds and amines, showed enhanced responses in several chicken lines, suggesting variation in the response patterns associated with sulfur- and nitrogen-containing volatiles. In contrast, sensor_3 and sensor_10 exhibited consistently weak responses with limited variation among chicken lines, implying that the volatile compounds associated with these sensors contributed less to the discrimination of odor profiles. Furthermore, sensor_9, which is highly sensitive to aromatic compounds, maintained relatively strong responses across all chicken lines, although noticeable variation among lines was still observed, indicating differences in the relative response patterns associated with aromatic volatiles.

### 3.2. Line-Associated Variation in Volatile Compounds Revealed by HS-SPME-GC–MS

#### 3.2.1. Volatile Compounds Identified by HS-SPME-GC–MS

Headspace solid-phase microextraction coupled with gas chromatography–mass spectrometry (HS-SPME-GC–MS) was employed to characterize the volatile organic compounds (VOCs) in Zao-Po vinegar Wenchang chicken prepared from different Wenchang chicken lines. A total of 85 VOCs were identified and classified into 11 chemical categories, including 4 acids, 15 alcohols, 10 aldehydes, 11 esters, 5 furans and furanones, 15 hydrocarbons and other compounds, 9 ketones, 1 lactone, 2 nitrogen-containing heterocyclic compounds, 10 sulfur-containing compounds, and 3 terpenes (Table S1). Among these, alcohols, esters, aldehydes, ketones, and sulfur-containing compounds constituted the major volatile constituents, comprising 55 compounds of the total identified VOCs.

These volatile compounds are likely derived from multiple pathways, including lipid oxidation, Maillard reactions, degradation of sulfur-containing precursors, esterification reactions, and fermentation-related metabolic processes. The combined contributions of these pathways are considered to shape the complex and distinctive volatile profile of Zao-Po vinegar Wenchang chicken [4,12,13,14].

The aldehydes mainly included pentanal, heptanal, octanal, nonanal, decanal, and undecanal, which are typically generated through the oxidation of unsaturated fatty acids and have been reported to exhibit fatty, green, fruity, and citrus-like odor characteristics [15]. Representative ketones included 2,3-octanedione, 2,5-octanedione, 4-octanone, and 3-butyl-2-heptanone, which have been reported to exhibit buttery, fatty, and roasted odor characteristics in food systems [4]. Alcohols were another important group of VOCs detected in the samples, including 1-octen-3-ol, 1-nonen-3-ol, and 2-nonen-1-ol. Among them, 1-octen-3-ol, a lipid-derived unsaturated alcohol commonly known as “mushroom alcohol”, has been reported to exhibit mushroom-like, green, fatty, and oily odor characteristics in cooked meat and mushroom products. Other unsaturated alcohols, including 1-nonen-3-ol and 2-nonen-1-ol, have also been associated with green and lipid-derived odor characteristics [16]. Esters, including ethyl formate, pentyl hexanoate, allyl 2-ethylbutyrate, and geranyl isovalerate, have been reported to exhibit fruity, sweet, and floral odor characteristics [17]. In addition, several sulfur-containing compounds, including diallyl sulfide, methyl allyl disulfide, diallyl tetrasulfide, and 5,6-dimethyl-6-hydroxytetrahydro-1,3-thiazine-2-thione, were detected. Sulfur-containing volatile compounds have been reported to exhibit low odor thresholds and characteristic sulfurous, garlic-like, onion-like, fermented, and cooked-meat odor notes in various food systems [13,16].

Although similar VOC classes were detected across all Wenchang chicken lines, noticeable differences in the abundance of individual compounds were observed. Therefore, subsequent statistical analyses were performed to identify VOCs showing significant line-associated differences.

#### 3.2.2. Characterization of Differential VOCs Among Wenchang Chicken Lines

To evaluate line-associated differences in individual VOCs, VOC abundance data were log10-transformed and analyzed using Welch’s one-way ANOVA, which does not assume equal variances among groups. To account for multiple testing across the 85 identified VOCs, the resulting *p* values were adjusted using the Benjamini–Hochberg false discovery rate (FDR) procedure. Among the 85 VOCs, 14 showed significant overall differences among Wenchang chicken lines after FDR correction (FDR-adjusted *p* < 0.05; Table 1). These included five aldehydes, two esters, two ketones, two sulfur-containing compounds, two furan/furanone-related compounds, and one other compound, indicating that line-associated variation in volatile profiles involved multiple chemical classes.

Values are expressed as mean ± SD (n = 3 biological replicates per line) on the original measurement scale. Overall differences among lines were assessed using Welch’s one-way ANOVA on log10-transformed VOC abundance data. To account for multiple testing across the 85 identified VOCs, Welch’s ANOVA *p* values were adjusted using the Benjamini–Hochberg false discovery rate (FDR) procedure. Only VOCs with FDR-adjusted *p* < 0.05 are presented. For these VOCs, pairwise comparisons among chicken lines were performed using the Games–Howell post-hoc test. Different superscript letters within the same row indicate significant pairwise differences (*p* < 0.05); means sharing at least one common superscript letter are not significantly different. Complete statistical results for all 85 identified VOCs are provided in Table S1. Pentanal showed relatively high mean abundances in Y and U3, whereas several aldehydes, including heptanal, octanal, nonanal, and decanal, showed relatively high mean abundances in Z2. Among the sulfur- and furan-related compounds, 5,6-dimethyl-6-hydroxytetrahydro-1,3-thiazin-2-thione showed relatively high mean abundances in U1 and U2, whereas 2-furanmethanol, tetrahydro-5-methyl-, trans- was relatively abundant in U1. The ketones 2,3-octanedione and 4-octanone also differed among lines. Overall, these results demonstrate significant line-associated variation in the abundance of individual VOCs in Zao-Po vinegar Wenchang chicken.

#### 3.2.3. Accumulation Patterns of Differential VOCs Among Wenchang Chicken Lines

To further visualize the line-associated variation in the 14 differential VOCs selected based on FDR-adjusted *p* < 0.05 cut-off, a hierarchical clustering heatmap was generated based on their mean abundances across the 12 Wenchang chicken lines (Figure 3). Distinct accumulation patterns were observed among the chicken lines, indicating marked line-associated variation in the relative abundances of these differential VOCs.

Several VOCs showed distinct line-associated accumulation patterns. Z2 exhibited relatively higher standardized abundances of several aldehydes, particularly heptanal, octanal, nonanal, and decanal, whereas pentanal showed relatively higher standardized abundances in Y, U3, and J. Other differential VOCs displayed distinct line-associated accumulation patterns. Ethyl formate showed a particularly high standardized abundance in U3, while acetic acid, hydroxy-, ethyl ester showed a relatively high standardized abundance in Z1. Among the sulfur- and furan-related compounds, 5,6-dimethyl-6-hydroxytetrahydro-1,3-thiazin-2-thione showed a relatively high standardized abundance in U2, 5-methyl-1,2,3,4-tetrathiane in T, 3(2H)-furanone, dihydro-5-isopropyl- in U4, and 2-furanmethanol, tetrahydro-5-methyl-, trans- in U1. The two ketones, 2,3-octanedione and 4-octanone, also exhibited distinct accumulation patterns across the chicken lines.

Hierarchical clustering further revealed similarities in the accumulation patterns of both VOCs and chicken lines. Several aldehydes, including heptanal, octanal, nonanal, and decanal, clustered relatively closely, indicating broadly similar patterns of variation across lines. At the chicken-line level, B and W clustered closely, as did U3 and Y, whereas the remaining lines showed distinct clustering relationships and combinations of differential VOCs. Collectively, these results indicate that line-associated differences in volatile profiles were characterized by distinct accumulation patterns involving multiple classes of VOCs rather than variation in a single compound.

### 3.3. Line-Associated Variation in Volatile Fingerprints Revealed by HS-GC–IMS

#### Characterization of Volatile Compounds by HS-GC–IMS

To further characterize line-associated variation in volatile profiles among Wenchang chicken lines, volatile fingerprints were analyzed using the Gallery Plot module of HS-GC–IMS (Figure 4). A total of 25 characteristic signal peaks, corresponding to 23 volatile compounds, were identified, including four aldehydes, three alcohols, seven esters, two ketones, three sulfur-containing compounds, one furan, one acid, one nitrile, and one amide. Methyl isovalerate and propyl butanoate were each detected in both monomer (M) and dimer (D) forms, accounting for the difference between the number of signal peaks and unique compounds; therefore, the 25 signal peaks corresponded to 23 unique volatile compounds. Therefore, the monomer and dimer peaks represent different ionization forms of the same compound rather than different chemical substances.

Representative compounds detected by GC–IMS included hexanal, 2-hexenal, 3-methylbutanal, octanal, 1-octen-3-ol, 2-octanol, hexyl acetate, ethyl propionate, isobutyl propanoate, isopropyl butanoate, diallyl sulfide, diallyl disulfide, dipropyl disulfide, 2-pentylfuran, acetone, 2-cyclohexen-1-one, acetic acid, and N-ethylacetamide. These compounds have been associated in previous studies with pathways such as lipid oxidation, esterification, degradation of sulfur-containing precursors, and fermentation-related metabolism.

The Gallery Plot further revealed obvious differences in the distribution and signal intensities of volatile compounds among Wenchang chicken lines. Although all lines shared a generally similar volatile composition, noticeable variation was observed in several regions corresponding to aldehydes, esters, alcohols, and sulfur-containing compounds. In particular, hexanal, octanal, 1-octen-3-ol, hexyl acetate, methyl isovalerate (M/D), propyl butanoate (M/D), diallyl sulfide, diallyl disulfide, dipropyl disulfide, and 2-pentylfuran showed pronounced variation in signal intensity among chicken lines and were therefore representative of the line-associated differences observed in the GC–IMS volatile fingerprints.

Overall, HS-GC–IMS showed that, although the Wenchang chicken lines shared broadly similar volatile compound compositions, they exhibited distinct relative signal patterns for several VOCs. The HS-GC–IMS results provided complementary information to the HS-SPME-GC–MS analysis, with both platforms revealing line-associated variation involving several VOC classes, including aldehydes, alcohols, esters, and sulfur-containing compounds. Because no direct quantitative cross-platform comparison was performed, these observations should be interpreted as complementary rather than confirmatory.

### 3.4. Integrated Analysis of Volatile Compounds by HS-SPME-GC–MS and HS-GC–IMS

Integrated analysis of the volatile profiles obtained by HS-SPME-GC–MS and HS-GC–IMS revealed both common overall characteristics and line-associated variation among the twelve Wenchang chicken lines. HS-SPME-GC–MS identified 85 VOCs belonging to 11 chemical classes, whereas HS-GC–IMS detected 25 characteristic signal peaks corresponding to 23 volatile compounds. The compounds detected by the two techniques covered several similar chemical classes, particularly aldehydes, alcohols, ketones, esters, and sulfur-containing compounds, although differences were observed in the range of compounds detected by each analytical platform.

HS-SPME-GC–MS provided broader coverage of the volatile composition and revealed significant line-associated differences in the abundance of specific VOCs. After correction for multiple comparisons using the Benjamini–Hochberg false discovery rate, 14 of the 85 VOCs remained significantly different among the twelve chicken lines (q < 0.05). These included several aldehydes, such as pentanal, heptanal, octanal, nonanal, and decanal, as well as ketones, esters, furan derivatives, and sulfur-containing compounds. These results indicated that line-associated variation was more evident in the abundance of specific VOCs than in the overall classes of volatile compounds detected.

HS-GC–IMS further revealed distinct line-associated patterns in volatile fingerprints (Figure 4). As shown in the Gallery Plot, the twelve chicken lines shared many characteristic signal peaks, but substantial differences in signal intensity were observed for several compounds across lines. The regions highlighted in the Gallery Plot illustrate groups of volatile signals with relatively high or low intensities in particular chicken lines, indicating heterogeneity in the distribution of volatile fingerprints among the samples. Thus, although the overall volatile composition was broadly similar among lines, the relative signal patterns of individual compounds differed considerably.

Overall, HS-SPME-GC–MS and HS-GC–IMS provided complementary information for characterizing the volatile profiles of Zao-Po vinegar Wenchang chicken. HS-SPME-GC–MS offered broader compound coverage and quantitative comparison of individual VOCs, whereas HS-GC–IMS provided intuitive visualization of line-associated differences in volatile fingerprints. Taken together, the two analytical approaches provided consistent and complementary evidence that the twelve Wenchang chicken lines shared broadly similar classes of volatile compounds but differed in the abundance and signal patterns of individual VOCs under standardized processing conditions.

### 3.5. Biochemical and Antioxidant Characteristics and Correlation Analysis with Volatile Compounds

#### 3.5.1. Biochemical and Antioxidant Characteristics of Different Wenchang Chicken Lines

The biochemical and antioxidant parameters varied among different Wenchang chicken lines (Figure 5). One-way analysis of variance (ANOVA) revealed significant differences (*p* < 0.05) in albumin (ALB), aspartate aminotransferase (AST), total cholesterol (CHO), glucose (GLU), high-density lipoprotein cholesterol (HDL), total protein (TP), malondialdehyde (MDA), and total antioxidant capacity (T-AOC) among the chicken lines, indicating line-associated variation in biochemical and antioxidant characteristics.

Among these indices, ALB and TP, which reflect protein nutritional status, differed significantly among the chicken lines. Similarly, CHO and HDL, which are associated with lipid metabolism, also exhibited pronounced line-dependent variation. In addition, significant differences in MDA and T-AOC were observed, suggesting variation in oxidative stress status and antioxidant capacity among different Wenchang chicken lines.

#### 3.5.2. Correlation Analysis Between Biochemical and Antioxidant Parameters and Volatile Compounds

To examine the associations between biochemical and antioxidant characteristics and volatile profiles among different Wenchang chicken lines, Pearson correlation analysis was performed between biochemical and antioxidant parameters and volatile organic compounds (VOCs) using line-level mean values (n = 12 lines). *p* values were adjusted for multiple comparisons using the Benjamini–Hochberg false discovery rate (BH-FDR) procedure.

After BH-FDR correction, 27 biochemical/antioxidant parameter–VOC correlations remained significant (FDR < 0.05; Figure 6). All significant correlations were positive and were restricted to CHO, TP, and MDA. CHO showed significant positive correlations with nine VOCs, including undecanal, hexanoic acid pentyl ester, hexanoic acid octyl ester, 2-nonen-1-ol, γ-dodecalactone, disulfide methyl 2-propenyl, cis-3,5-diethyl-1,2,4-trithiolane, phytol, and acetic acid hydroxy ethyl ester (r = 0.912–0.920, FDR = 0.0015–0.0022).

TP and MDA showed similar correlation patterns. TP was positively correlated with the same nine VOCs (r = 0.830–0.861, FDR = 0.0068–0.0165), while MDA was also positively correlated with these nine compounds (r = 0.860–0.861, FDR = 0.0068). In contrast, none of the correlations involving ALB, AST, GLU, HDL, or T-AOC remained significant after FDR correction (FDR ≥ 0.05).

Overall, the FDR-adjusted analysis identified a restricted set of significant associations between CHO, TP, MDA, and nine VOCs. Because the analysis was based on line-level mean values (n = 12 lines), these results represent statistical associations and should not be interpreted as evidence of causal relationships between biochemical or antioxidant characteristics and VOC formation.

## 4. Discussion

### 4.1. Line-Associated Variation in Volatile Flavor Profiles Among Wenchang Chicken Lines

Under standardized rearing and Zao-Po vinegar processing conditions, E-nose, HS-SPME-GC–MS, and HS-GC–IMS analyses consistently revealed line-associated variation in the volatile profiles of the twelve Wenchang chicken lines. The lines shared broadly similar classes of VOCs, whereas their relative abundances and signal patterns differed. Previous studies have similarly reported breed- or line-associated differences in intramuscular fat, fatty acid composition, amino acid profiles, and other traits related to flavor precursor availability in chicken meat [4,18].

The Wenchang chicken lines examined in this study have different selection histories and breeding objectives, which may be associated with biological differences among lines. Such differences could potentially influence the availability of flavor precursors and their subsequent transformation during cooking. However, because no direct genetic analysis or measurement of muscle flavor precursors was performed, the present results do not establish a genetic mechanism underlying the observed volatile differences. We therefore hypothesize that variation in precursor composition and availability may be one factor contributing to the observed line-associated volatile profiles.

### 4.2. Multiple Biochemical Pathways Potentially Contribute to the Volatile Profile of Zao-Po Vinegar Wenchang Chicken

The VOCs detected in Zao-Po vinegar Wenchang chicken included aldehydes, alcohols, ketones, esters, sulfur-containing compounds, and other chemical classes, suggesting contributions from multiple biochemical and processing-related pathways. Aldehydes and alcohols such as nonanal, decanal, undecanal, and 1-octen-3-ol are commonly associated with lipid oxidation, whereas ketones and sulfur-containing compounds may arise from lipid degradation, Maillard reactions, Strecker degradation, and reactions involving sulfur-containing precursors [4,18,19,20].

Zao-Po vinegar may provide additional fermentation-derived components, including organic acids and other metabolites, that can interact with endogenous meat-derived precursors during thermal processing [21]. Thus, the observed volatile profile likely reflects interactions among lipid oxidation, thermal reactions, and fermentation-derived components. These pathways represent plausible explanations for the observed VOC patterns rather than mechanisms that were directly demonstrated in the present study.

### 4.3. Associations Between Biochemical Characteristics and Volatile Profiles Among Wenchang Chicken Lines

After BH-FDR correction, significant correlations with VOCs were restricted to CHO, TP, and MDA, whereas correlations involving ALB, AST, GLU, HDL, and T-AOC did not remain significant. CHO and TP are serum biochemical indicators that may provide indirect information related to lipid and protein metabolic status, respectively, while MDA is commonly used as an indicator of lipid peroxidation [22,23,24]. Their associations with specific VOCs therefore suggest coordinated line-level variation between physiological status and volatile composition.

Notably, MDA, a well-established indicator of lipid peroxidation, remained positively associated with a subset of VOCs after FDR correction, including 2-nonen-1-ol and undecanal [25]. This association is consistent with a potential relationship between oxidative status and variation in volatile composition. Moreover, lipid oxidation products may interact with Maillard reaction intermediates, thereby contributing to the complexity of VOC formation during thermal processing [20,26].

However, serum biochemical parameters do not directly measure flavor precursors or reaction products in muscle, and the present correlations were based on line-level means (n = 12). Consequently, these associations should not be interpreted as evidence of causal mechanisms of VOC formation. Future studies incorporating direct measurements of muscle fatty acid composition, free amino acids, lipid oxidation products, metabolomics, lipidomics, and sensory evaluation will be necessary to test the proposed relationships and clarify their biological basis.

### 4.4. Integrated Analytical Strategy Provides Complementary Characterization of Volatile Profiles

By integrating E-nose, HS-SPME-GC–MS, HS-GC–IMS, and biochemical analyses, this study characterized line-associated variation in Zao-Po vinegar Wenchang chicken from complementary perspectives, including overall odor fingerprints, volatile composition, and physiological status. E-nose analysis enabled rapid characterization of overall odor fingerprints, HS-SPME-GC–MS provided broad characterization of VOCs, HS-GC–IMS provided complementary information on volatile fingerprints, and biochemical measurements provided additional information on line-level physiological variation. Collectively, this integrated analytical strategy provides a basis for the comparative characterization of Wenchang chicken lines and for future studies investigating the biological basis and sensory relevance of line-associated variation in volatile profiles.

## 5. Conclusions

This study provides an integrated characterization of line-associated variation in the volatile profiles of Zao-Po vinegar Wenchang chicken from twelve Wenchang chicken lines under standardized rearing and processing conditions. E-nose analysis revealed clear differences in overall odor response patterns among the lines, while HS-SPME-GC–MS identified 85 VOCs across 11 chemical classes, with 14 compounds retaining significant line-associated differences after BH-FDR correction. HS-GC–IMS detected 25 characteristic signal peaks corresponding to 23 unique volatile compounds and provided complementary information on volatile fingerprints. Together, these analytical platforms consistently demonstrated that the Wenchang chicken lines share broadly similar classes of volatile compounds but differ in the relative abundance and distribution patterns of individual VOCs.

The correlation analysis further identified FDR-supported associations between selected biochemical and antioxidant parameters and volatile composition, with significant associations restricted to CHO, TP, and MDA. These findings suggest coordinated line-level variation between physiological characteristics and volatile profiles; however, because serum biochemical parameters are indirect indicators and the correlation analysis was based on line-level means, these associations should not be interpreted as evidence of causal mechanisms for VOC formation. Overall, the present study establishes a comprehensive analytical framework for comparing the volatile characteristics of Zao-Po vinegar Wenchang chicken across different lines and provides a basis for further investigation of the biological factors underlying line-associated variation in volatile profiles. Future studies integrating direct measurements of muscle fatty acids, free amino acids, lipid oxidation products, metabolomics and lipidomics with sensory evaluation will be important for determining the biological basis and sensory relevance of these differences.

## Figures and Tables

**Figure 1:**
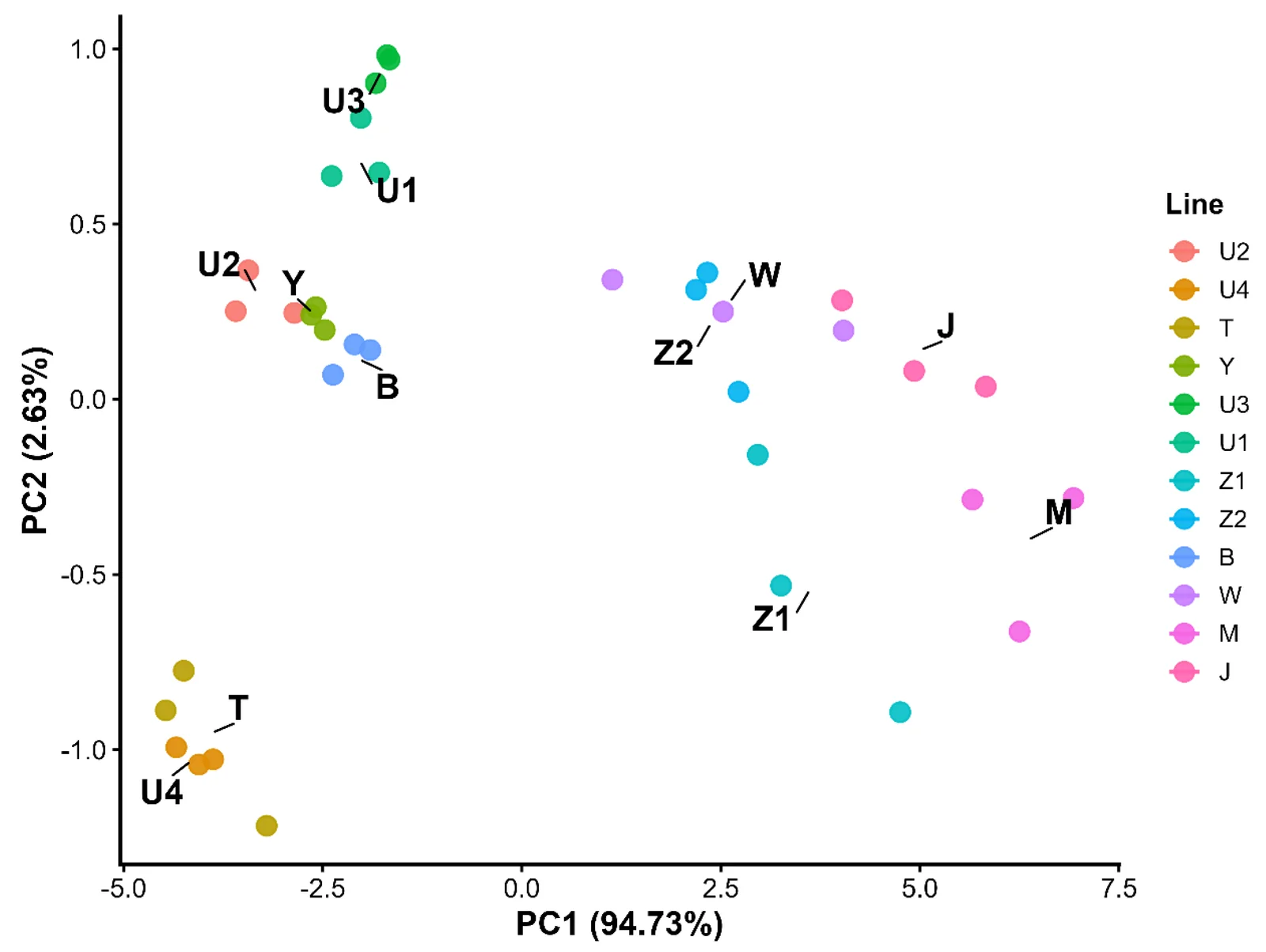
PCA plot of E-nose analysis of Zao-Po vinegar chicken made from different Wenchang chicken lines.

**Figure 2:**
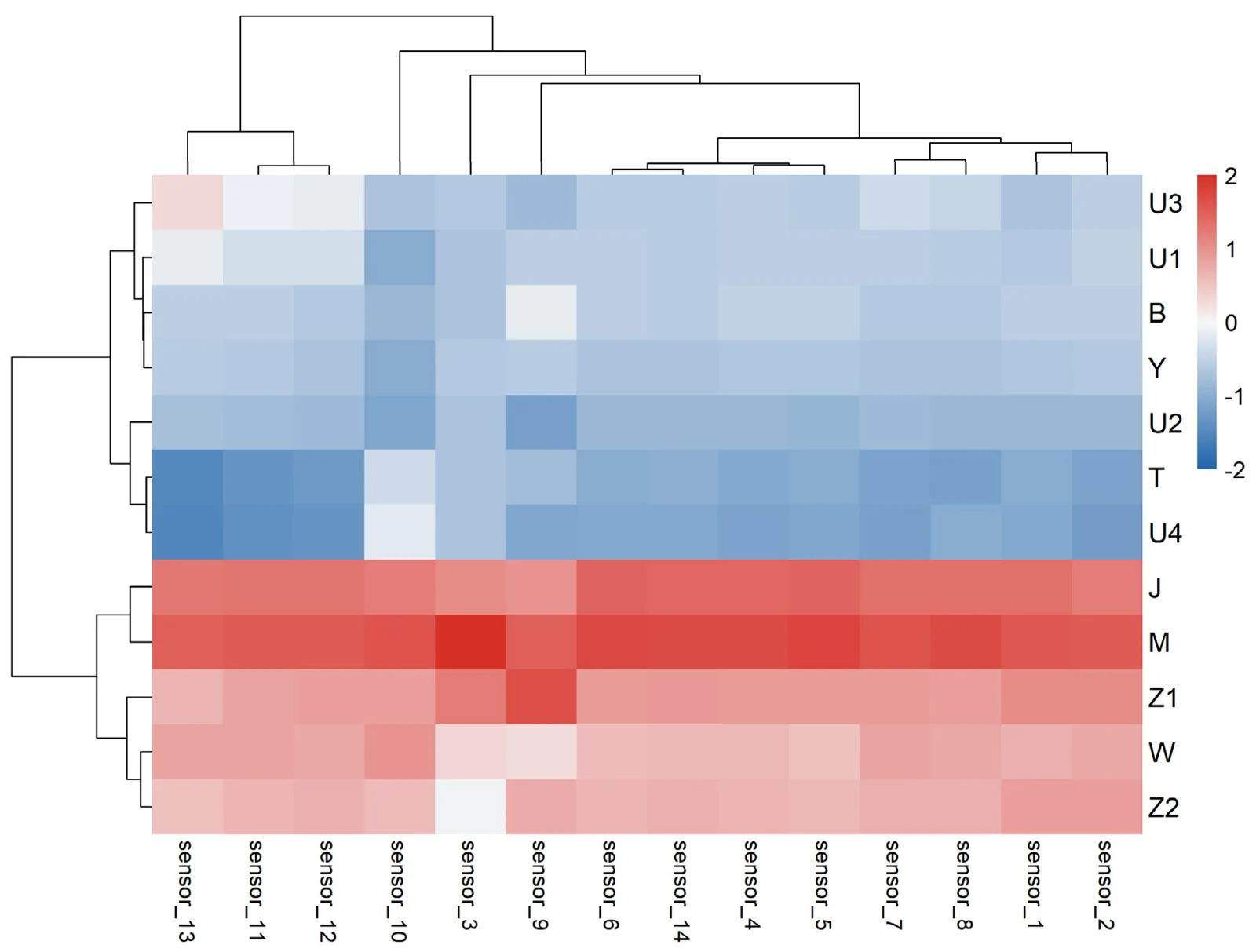
HCA heatmap of E-nose analysis of Zao-Po vinegar chicken made from different Wenchang chicken lines.

**Figure 3:**
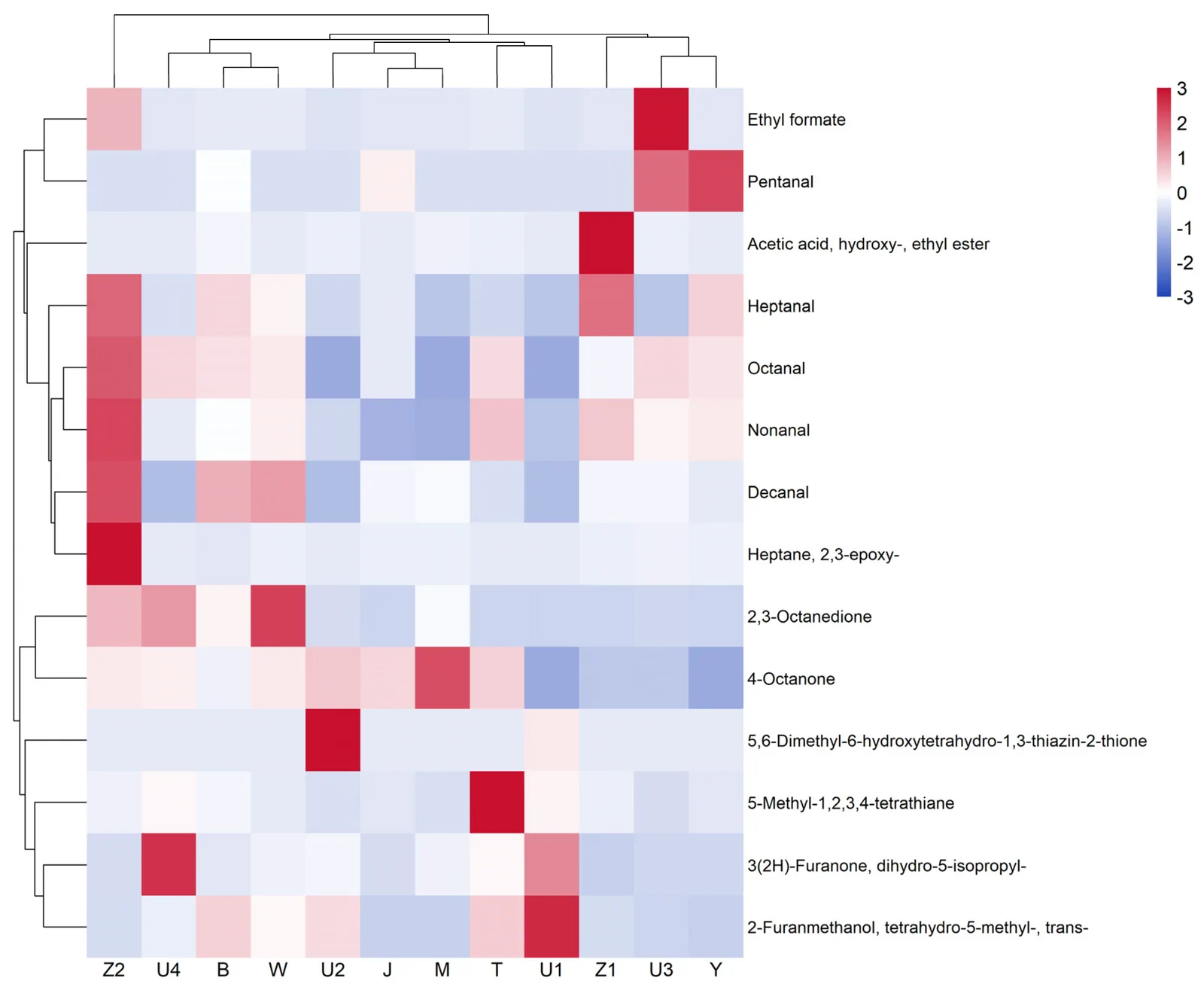
Hierarchical clustering heatmap of the 14 differential volatile organic compounds (VOCs) among Wenchang chicken lines. The VOCs were selected based on FDR-adjusted *p* < 0.05. Mean abundances of three biological replicates per line were subjected to row-wise Z-score normalization. Red and blue indicate relatively higher and lower standardized abundances, respectively, for each VOC across chicken lines. Hierarchical clustering was performed using Euclidean distance and complete linkage.

**Figure 4:**
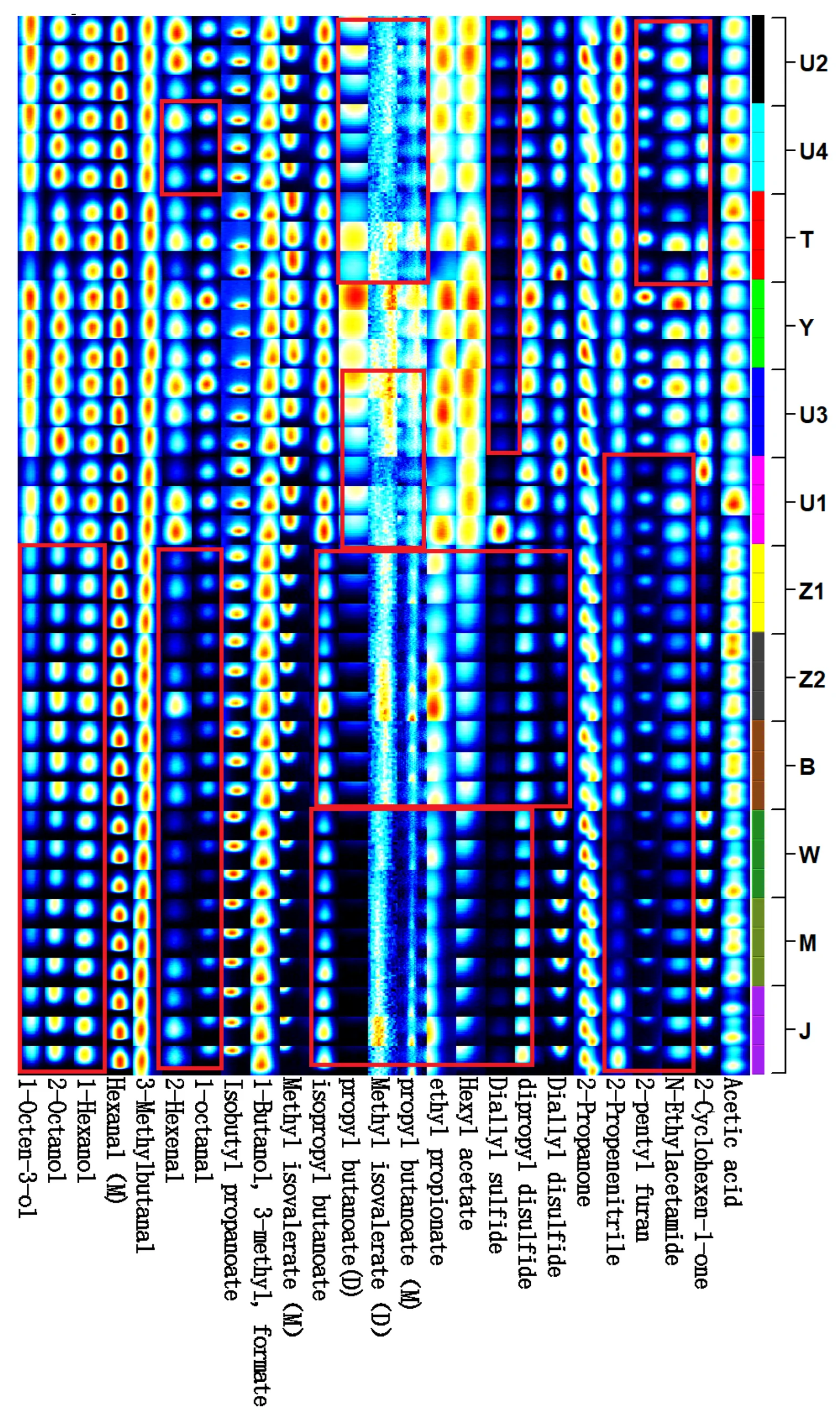
HS-GC–IMS gallery plot showing the volatile fingerprints of Zao-Po vinegar Wenchang chicken prepared from different Wenchang chicken lines. Each row represents an individual sample, and each column represents a characteristic GC–IMS signal peak. Signal intensity is indicated by color, with warmer colors representing higher signal intensities.

**Figure 5:**
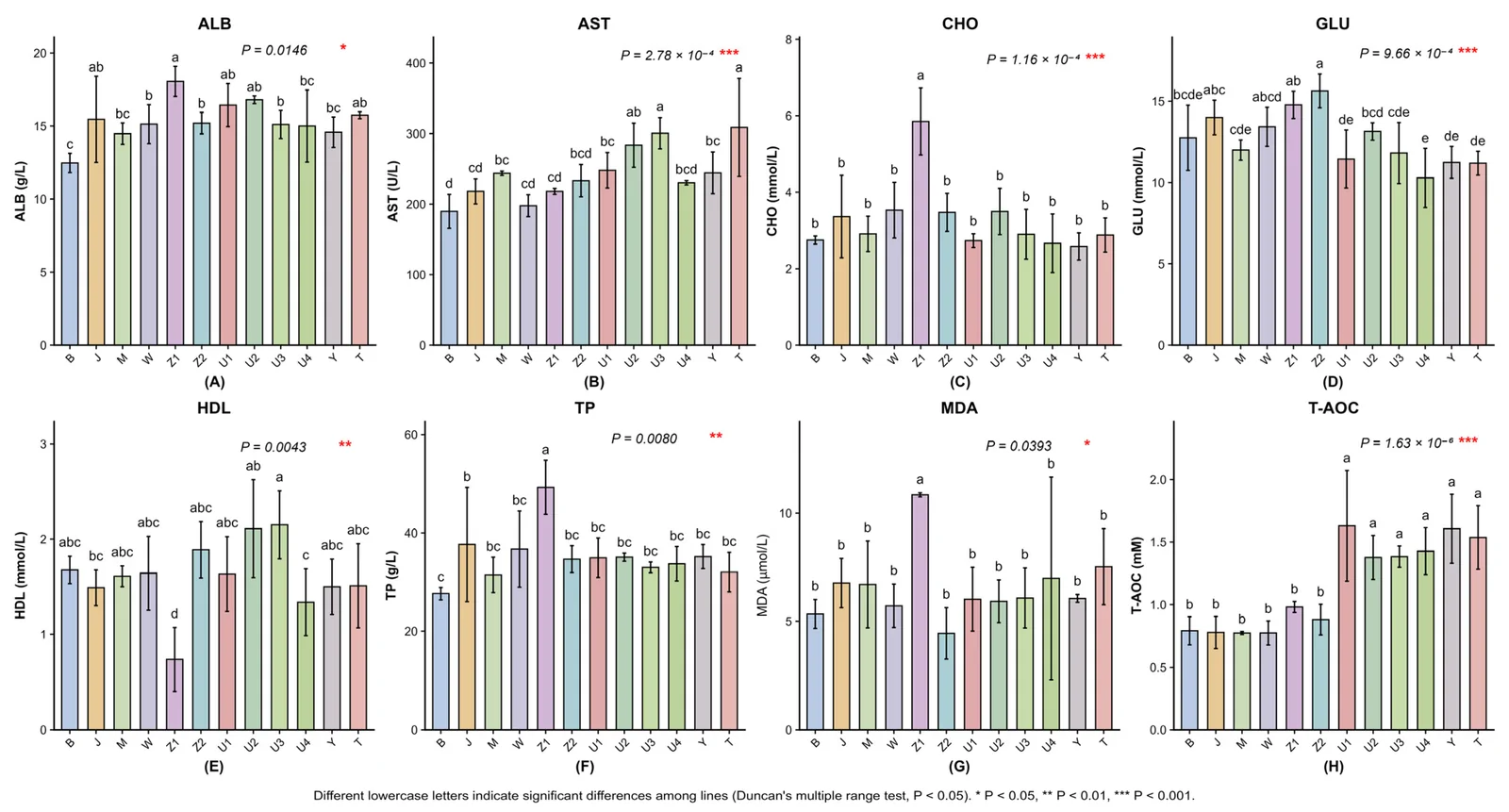
Biochemical and antioxidant parameters of different Wenchang chicken lines. (**A**) Albumin (ALB); (**B**) aspartate aminotransferase (AST); (**C**) total cholesterol (CHO); (**D**) glucose (GLU); (**E**) high-density lipoprotein cholesterol (HDL); (**F**) total protein (TP); (**G**) malondialdehyde (MDA); and (**H**) total antioxidant capacity (T-AOC).

**Figure 6:**
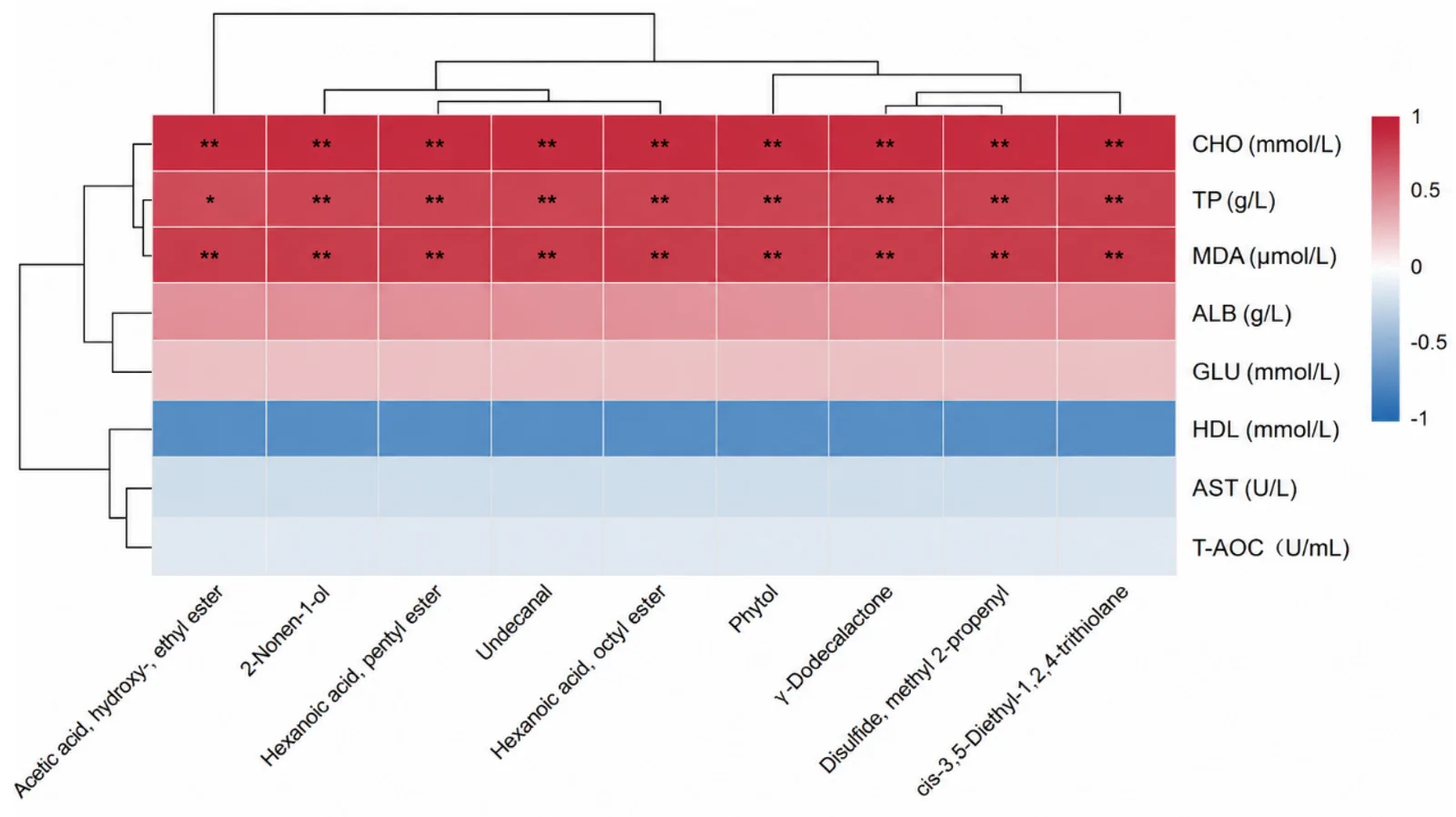
Pearson correlation heatmap between biochemical and antioxidant parameters and volatile compounds identified by HS-SPME-GC–MS in Zao-Po vinegar Wenchang chicken. Only VOCs showing at least one significant correlation with a biochemical or antioxidant parameter after BH-FDR correction (FDR < 0.05) are displayed, while all measured biochemical and antioxidant parameters are retained. Correlations were calculated using line-level mean values (n = 12 lines). Red and blue indicate positive and negative correlations, respectively, and color intensity represents the magnitude of the Pearson correlation coefficient (r). *p* values were adjusted for multiple comparisons using the Benjamini–Hochberg false discovery rate (FDR) procedure. Asterisks indicate correlations that remained significant after FDR correction (* FDR < 0.05, ** FDR < 0.01). Hierarchical clustering was performed for both biochemical and antioxidant parameters and volatile compounds.

**Table 1:** Volatile organic compounds showing significant line-associated differences among Wenchang chicken lines after FDR correction. Different lowercase letters within the same row indicate significant differences among lines (FDR-adjusted *p* < 0.05), and values sharing the same letter are not significantly different.

CAS	Compound Name	Molecular Formula	B	J	M	T	U1	U2	U3	U4	W	Y	Z1	Z2	Welch_P	FDR
124-13-0	Octanal	C8H16O	14.929 ± 2.875 a	8.811 ± 4.362 ab	0.277 ± 0.037 bc	15.012 ± 17.169 abc	0.246 ± 0.087 c	0.265 ± 0.086 c	15.465 ± 2.011 a	15.816 ± 2.232 a	13.939 ± 3.368 a	14.091 ± 0.684 a	10.141 ± 4.312 a	28.557 ± 16.320 a	4.09 × 10^−9^	3.48 × 10^−7^
110-62-3	Pentanal	C5H10O	30.374 ± 51.794 ab	45.164 ± 78.171 ab	0.050 ± 0.005 b	0.580 ± 0.974 ab	0.053 ± 0.037 b	0.026 ± 0.019 b	145.476 ± 76.915 a	0.636 ± 1.045 ab	0.060 ± 0.015 b	177.830 ± 61.986 a	0.328 ± 0.451 ab	0.067 ± 0.020 b	1.35 × 10^−7^	5.74 × 10^−6^
112-31-2	Decanal	C10H20O	6.508 ± 1.765 ab	2.926 ± 0.734 ab	3.016 ± 2.569 abcd	1.802 ± 1.433 abcd	0.083 ± 0.043 c	0.124 ± 0.083 abcd	2.936 ± 0.612 b	0.089 ± 0.045 cd	7.333 ± 1.807 ab	2.332 ± 0.234 bd	2.992 ± 0.975 ab	10.324 ± 0.447 a	5.70 × 10^−7^	1.61 × 10^−5^
109-94-4	Ethyl formate	C3H6O2	3.372 ± 0.438 a	0.814 ± 1.406 ab	1.768 ± 2.267 ab	3.907 ± 5.713 ab	0.001 ± 0.000 b	0.001 ± 0.001 b	168.512 ± 287.596 ab	0.303 ± 0.524 ab	3.494 ± 3.365 ab	1.835 ± 2.997 ab	1.913 ± 3.250 ab	68.643 ± 118.889 ab	1.10 × 10^−6^	1.87 × 10^−5^
54774-28-6	2-Furanmethanol, tetrahydro-5-methyl-, trans-	C6H12O2	0.303 ± 0.520 abc	0.002 ± 0.002 bc	0.002 ± 0.001 b	0.314 ± 0.543 abc	0.771 ± 0.028 a	0.280 ± 0.481 abc	0.014 ± 0.023 abc	0.108 ± 0.182 abc	0.189 ± 0.043 c	0.002 ± 0.001 b	0.049 ± 0.039 abc	0.043 ± 0.069 abc	9.80 × 10^−7^	1.87 × 10^−5^
585-25-1	2,3-Octanedione	C8H14O2	15.731 ± 26.221 abc	0.050 ± 0.084 abc	11.253 ± 17.834 abc	0.517 ± 0.410 ab	0.002 ± 0.002 c	2.828 ± 4.407 abc	1.286 ± 0.430 a	36.315 ± 5.260 b	55.521 ± 49.895 abc	0.014 ± 0.021 abc	0.345 ± 0.270 abc	28.571 ± 49.433 abc	6.75 × 10^−6^	9.56 × 10^−5^
34004-69-8	3(2H)-Furanone, dihydro-5-isopropyl-	C7H12O2	0.338 ± 0.580 abc	0.187 ± 0.193 abc	0.541 ± 0.220 ab	0.831 ± 0.649 ab	2.081 ± 0.781 a	0.597 ± 0.809 abc	0.147 ± 0.162 abc	3.116 ± 5.208 abc	0.543 ± 0.709 abc	0.135 ± 0.029 b	0.003 ± 0.001 c	0.183 ± 0.312 abc	1.03 × 10^−5^	1.25 × 10^−4^
111-71-7	Heptanal	C7H14O	6.157 ± 2.941 a	2.555 ± 1.773 ab	0.131 ± 0.187 abc	1.327 ± 2.248 abc	0.015 ± 0.008 c	1.424 ± 2.437 abc	0.019 ± 0.013 bc	1.932 ± 3.299 abc	4.516 ± 4.206 abc	6.403 ± 6.225 abc	11.624 ± 17.227 abc	11.903 ± 8.771 a	9.23 × 10^−5^	9.81 × 10^−4^
116664-30-3	5-Methyl-1,2,3,4-tetrathiane	C3H6S4	0.080 ± 0.003 a	0.055 ± 0.037 ab	0.039 ± 0.017 ab	0.424 ± 0.615 ab	0.109 ± 0.002 b	0.041 ± 0.010 ab	0.036 ± 0.023 ab	0.107 ± 0.002 b	0.058 ± 0.028 ab	0.053 ± 0.034 ab	0.066 ± 0.043 ab	0.075 ± 0.028 ab	2.45 × 10^−4^	2.31 × 10^−3^
124-19-6	Nonanal	C9H18O	31.790 ± 6.847 ab	18.245 ± 5.290 ab	17.178 ± 17.853 ab	40.188 ± 10.999 ab	21.279 ± 4.540 ab	25.020 ± 3.607 b	33.494 ± 4.399 ab	28.467 ± 6.545 ab	34.465 ± 11.464 ab	35.162 ± 1.762 b	39.648 ± 11.109 ab	57.697 ± 3.351 a	3.17 × 10^−4^	2.69 × 10^−3^
135923-17-0	5,6-Dimethyl-6-hydroxytetrahydro-1,3-thiazin-2-thione	C6H11NOS2	0.023 ± 0.006 a	0.040 ± 0.010 a	0.035 ± 0.021 a	0.047 ± 0.026 a	1.829 ± 0.857 b	9.960 ± 10.719 ab	0.029 ± 0.008 a	0.039 ± 0.012 a	0.046 ± 0.007 a	0.055 ± 0.025 a	0.031 ± 0.019 a	0.033 ± 0.028 a	1.00 × 10^−3^	7.08 × 10^−3^
589-63-9	4-Octanone	C8H16O	0.360 ± 0.464 ab	0.597 ± 0.490 ab	1.136 ± 1.198 a	0.612 ± 0.609 ab	0.003 ± 0.002 b	0.658 ± 1.135 ab	0.150 ± 0.253 ab	0.506 ± 0.435 ab	0.515 ± 0.451 a	0.002 ± 0.001 b	0.152 ± 0.258 ab	0.513 ± 0.457 ab	1.00 × 10^−3^	7.08 × 10^−3^
623-50-7	Acetic acid, hydroxy-, ethyl ester	C4H8O3	1.862 ± 1.476 a	0.004 ± 0.002 b	1.284 ± 2.137 ab	0.917 ± 1.578 ab	0.005 ± 0.001 b	0.659 ± 0.650 ab	0.599 ± 1.030 ab	0.005 ± 0.004 b	0.004 ± 0.002 b	0.060 ± 0.089 ab	33.083 ± 48.983 ab	0.008 ± 0.001 b	2.00 × 10^−3^	1.31 × 10^−2^
14925-96-3	Heptane, 2,3-epoxy-	C7H14O	0.001 ± 0.000 ab	0.003 ± 0.000 a	0.003 ± 0.001 ab	0.002 ± 0.001 ab	0.002 ± 0.000 b	0.002 ± 0.001 ab	0.003 ± 0.001 ab	0.002 ± 0.001 ab	0.003 ± 0.001 ab	0.002 ± 0.000 ab	0.003 ± 0.001 ab	0.065 ± 0.073 ab	5.00 × 10^−3^	3.04 × 10^−2^

## Data Availability

The original contributions presented in this study are included in the article/Supplementary Materials. Further inquiries can be directed to the corresponding authors.

## Supplementary Materials

The following supporting information can be downloaded at: https://www.mdpi.com/article/10.3390/foods15193464/s1, Table S1. Volatile organic compounds identified by HS-SPME-GC–MS in Zao-Po vinegar Wenchang chicken from twelve different lines, with their CAS numbers, compound names, molecular formulas, and relative contents (mean ± standard deviation). Table S2: The 14 sensors constitute the sensor array of the electronic nose. Each sensor responds to a specific class of volatile compounds, and the combined response pattern was used to discriminate among Wenchang chicken lines. Table S3: Basic information of the 36 Wenchang chickens used in this study.

## References

[1] Pellattiero E., Tasoniero G., Cullere M., Gleeson E., Baldan G., Contiero B., Dalle Zotte A. (2020). Are Meat Quality Traits and Sensory Attributes in Favor of Slow-Growing Chickens?. Animals.

[2] Li L., Zhang G., Huang B., Ren L., Zheng A., Zhang J., Sun Y. (2015). Silica-based helical rod supporting guanosine compounds catalyzed asymmetric hetero-Diels–Alder reaction. Catal. Commun..

[3] Jiang S., Zhang J. (2026). Isolation and identification of lactic acid bacteria from Hainan Zao-po-Vinegar based on single-cell Raman sorting technology. J. Chin. Inst. Food Sci. Technol..

[4] Mottram D.S. (1998). Flavour formation in meat and meat products: A review. Food Chem..

[5] Mueller S., Kreuzer M., Siegrist M., Mannale K., Messikommer R.E., Gangnat I.D.M. (2018). Carcass and meat quality of dual-purpose chickens (Lohmann Dual, Belgian Malines, Schweizerhuhn) in comparison to broiler and layer chicken types. Poult. Sci..

[6] Sandercock D.A., Nute G.R., Hocking P.M. (2009). Quantifying the effects of genetic selection and genetic variation for body size, carcass composition, and meat quality in the domestic fowl (*Gallus domesticus*). Poult. Sci..

[7] Siekmann L., Janisch S., Wigger R., Urban J., Zentek J., Krischek C. (2018). Lohmann Dual: A dual-purpose chicken as an alternative to commercial broiler chicken? Aspects of meat quality, lipid oxidation, shear force and muscle structure. Eur. Poult. Sci..

[8] Shin D.-J., Jung Y., Kim D., Jo C., Nam K.-C., Lee J.-H., Choo H.-J., Jang A. (2024). Identification and comparison of aroma and taste-related compounds from breast meat of three breeds of Korean native chickens. Poult. Sci..

[9] Yang J., Qin K., Wang Q., Li S., Zhu Y., Yang X. (2025). Comparative analysis of amino acids and volatile compounds as markers in Lueyang black-boned chicken and Arbor Acres broilers. Poult. Sci..

[10] Jin Y., Cui H., Yuan X., Liu L., Liu X., Wang Y., Ding J., Xiang H., Zhang X., Liu J. (2021). Identification of the main aroma compounds in Chinese local chicken high-quality meat. Food Chem..

[11] (2021). Product of Geographical Indication—Wenchang Chicken.

[12] Ho C.W., Lazim A.M., Fazry S., Zaki U.K.H.H., Lim S.J. (2017). Varieties, production, composition and health benefits of vinegars: A review. Food Chem..

[13] Elmore J.S., Mottram D.S., Enser M., Wood J.D. (1999). Effect of the Polyunsaturated Fatty Acid Composition of Beef Muscle on the Profile of Aroma Volatiles. J. Agric. Food Chem..

[14] Meynier A., Mottram D.S. (1995). The effect of pH on the formation of volatile compounds in meat-related model systems. Food Chem..

[15] Rochat S., Chaintreau A. (2005). Carbonyl Odorants Contributing to the In-Oven Roast Beef Top Note. J. Agric. Food Chem..

[16] Sohail A., Al-Dalali S., Wang J., Xie J., Shakoor A., Asimi S., Shah H., Patil P. (2022). Aroma compounds identified in cooked meat: A review. Food Res. Int..

[17] Li N., Li G., Li A., Tao Y. (2023). Synergy Effect between Fruity Esters and Potential Odorants on the Aroma of Hutai-8 Rose Wine Revealed by Threshold, S-Curve, and σ–τ Plot Methods. J. Agric. Food Chem..

[18] Jayasena D.D., Ahn D.U., Nam K.C., Jo C. (2013). Flavour Chemistry of Chicken Meat: A Review. Asian-Australas J. Anim. Sci..

[19] Bueno M., Resconi V.C., Campo M.M., Cacho J., Ferreira V., Escudero A. (2011). Gas chromatographic–olfactometric characterisation of headspace and mouthspace key aroma compounds in fresh and frozen lamb meat. Food Chem..

[20] Calkins C.R., Hodgen J.M. (2007). A fresh look at meat flavor. Meat Sci..

[21] Stahnke L.H. (1994). Aroma components from dried sausages fermented with Staphylococcus xylosus. Meat Sci..

[22] He X.M., Carter D.C. (1992). Atomic structure and chemistry of human serum albumin. Nature.

[23] Beski S.S.M., Swick R.A., Iji P.A. (2015). Specialized protein products in broiler chicken nutrition: A review. Anim. Nutr..

[24] Zálešáková D., Novotný J., Řiháček M., Horáková L., Mrkvicová E., Šťastník O., Pavlata L. (2025). The blood biochemical parameters intervals and dynamics in modern broiler chickens. Vet. Anim. Sci..

[25] Resconi V.C., Escudero A., Campo M.M. (2013). The Development of Aromas in Ruminant Meat. Molecules.

[26] Zamora R., Hidalgo F. (2005). Coordinate contribution of lipid oxidation and Maillard reaction to nonenzymatic food browning. Crit. Rev. Food Sci. Nutr..

